# The implementation of large-scale genomic screening or diagnostic programmes: A rapid evidence review

**DOI:** 10.1038/s41431-022-01259-8

**Published:** 2022-12-14

**Authors:** Germán Andrés Alarcón Garavito, Thomas Moniz, Noémie Déom, Federico Redin, Amanda Pichini, Cecilia Vindrola-Padros

**Affiliations:** 1 Rapid Research Evaluation and Appraisal Lab (RREAL), University College London, 43-45 Foley Street, W1W 7TY London, UK; 2 Genomics England, London, UK

**Keywords:** Genetics research, Genetic testing, Genetic counselling, Translational research

## Abstract

Genomic healthcare programmes, both in a research and clinical context, have demonstrated a pivotal opportunity to prevent, diagnose, and treat rare diseases. However, implementation factors could increase overall costs and affect uptake. As well, uncertainties remain regarding effective training, guidelines and legislation. The purpose of this rapid evidence review was to draw together the available global evidence on the implementation of genomic testing programmes, particularly on population-based screening and diagnostic programmes implemented at the national level, to understand the range of factors influencing implementation. This review involved a search of terms related to genomics, implementation and health care. The search was limited to peer-reviewed articles published between 2017–2022 and found in five databases. The review included thirty articles drawing on sixteen countries. A wide range of factors was cited as critical to the successful implementation of genomics programmes. These included having policy frameworks, regulations, guidelines; clinical decision support tools; access to genetic counselling; and education and training for healthcare staff. The high costs of implementing and integrating genomics into healthcare were also often barriers to stakeholders. National genomics programmes are complex and require the generation of evidence and addressing implementation challenges. The findings from this review highlight that there is a strong emphasis on addressing genomic education and engagement among varied stakeholders, including the general public, policymakers, and governments. Articles also emphasised the development of appropriate policies and regulatory frameworks to govern genomic healthcare, with a focus on legislation that regulates the collection, storage, and sharing of personal genomic data.

## Introduction

There has been considerable progress in the field of genomic medicine with the increasing application of genomic technologies in healthcare to screen, diagnose, and treat diseases [1, 2]. Despite the rapidly evolving field of genomic research, its integration and implementation into healthcare has been slow and varies drastically between and within countries [3, 4]. The provision of genomic medicine has thus far been hindered by inconclusive evidence of the clinical utility of genomic information and the insufficient implementation science evidence [1, 2].

Implementation science enables us to understand the factors shaping the adoption of genomics and to identify tools that support its implementation into healthcare. While healthcare providers broadly support the adoption of genomic medicine, they are limited in their ability to do so due to the lack of genomics education and training, healthcare fragmentation and the scarcity of digital tools for genomics integrated with electronic health records (EHR). Moreover, there remains a lack of research focusing on macro-level factors such as health systems, health policies, financing, and generalisability of genomics programmes [5]. In order to identify and overcome the barriers to the adoption of genomics and ensure its effective and timely translation into clinical settings, implementation frameworks and outcome measures should be applied from the initial research stages through to the development and integration of genomic medicine. Once genomics programmes are integrated into healthcare settings, they should continue to be evaluated and adapted and adjusted as necessary to ensure sustainable implementation [5].

A number of countries have adopted genomic healthcare programmes at a national or regional level. The United Kingdom has government support to lead developments in genomic medicine, delivered through a national health service (NHS) [6]. Following the delivery of the 100,000 Genomes Project, whole genome sequencing (WGS) is now available along with other clinical genomic tests for rare diseases and cancers with equitable access through a national NHS Genomic Medicine Service [7]. This includes linking clinical care with a national de-identified research database of genomic and health data (with patient consent) to promote a learning healthcare system. Genomics England (GEL), in partnership with the NHS, is now designing and implementing a research study to explore the potential benefits and challenges of WGS in newborns [8].

The Australian Genomics Health Alliance is a national network of state-level genomic initiatives collaborating to translate approaches to genomics into standardised practice through clinical and laboratory research. This includes a focus on health policy, health economics, education and implementation science [9].

In France, The Plan France Médecine Génomique 2025 (PFMG 2025) aims to develop a national framework for big-genomic data. Leading French public research organisations participate, while France Génomique manages the programme’s technological aspects, including sequencing platforms and data analysis infrastructure. Patients’ electronic records are standardised, and regulatory frameworks are being developed. The PFMG 2025 aims include developing an economic model and processes for harmonising protocols and methods to support implementation into healthcare.

There are understandably variable approaches to genomics programmes in different countries due to differences in funding, infrastructure and variable approaches to evaluating their implementation. This can present a challenge to harmonizing the enablers and barriers to implementation, including common factors that may be relevant internationally.

While a variety of examples of national or regional genomic healthcare programmes exist, there is a recently emerging body of evidence regarding evaluation and implementation of these programmes, including factors acting as barriers and enablers with variability in the number, type and duration of these studies in different countries internationally. In order to inform the effective planning and design of future programmes, it is helpful to understand these implementation factors up-front. This evidence is crucial to bridging the gap between research findings and the systematic and sustainable uptake of genomics into clinical care. This rapid evidence review aims to identify these factors and discuss their implications for programme planning, practice, and policy and support programme design by considering these implementation factors up-front. We chose a rapid review approach because it best supported Genomic England need for a capture of the current knowledge, trends and gaps in the fast-moving field of genomics, to inform its future programmes.

## Methods

The review design was informed by guidance for rapid evidence reviews developed by Tricco et al. [10]. The review followed a phased approach, beginning with a broad search strategy and subsequently expanding with each search round. We followed the Preferred Reporting Items for Systematic Reviews and Meta-Analysis (PRISMA) statement to guide the review design and report the methods and findings [11]. Due to the rapid nature of the review (10 weeks), the questions and search strategy were targeted to identify relevant articles that could be analysed within the review timeframe. A protocol was developed before searching and is registered in the Open Science Framework (OSF) platform (https://osf.io/hkywe/).

### Search strategy

We identified search terms using a combination of free-text and controlled terms building on previous work and suggestions from employees at GEL, as this review was commissioned to inform implementation approaches for programmes and services developed by GEL and delivered within the UK NHS.

We tested and refined the terms by running exploratory searches in principal databases. We assessed a provisional search strategy for sensitivity versus breadth on PubMed, using different combinations of Boolean operators and search strings (see Appendix 1 for the complete search strategy).

The search was limited to articles published between 2017 and 2022 due to the rapidly changing nature of genomic technologies and to keep the review scope manageable for a rapid timeframe. However, there was no language or location limitation. The search strategy focused on three categories (genomics, implementation science, and healthcare). Final searches were conducted in February 2022 on five databases (Web of Science, Medline, PubMed, CINAHL and EMBASE).

### Document selection

The search results were imported into Rayyan, which is a validated tool with semi-automated features enabling the detection of duplicated publications from the different databases [12]. The software also displays citation details, titles, and abstracts of each publication, facilitating screening.

The initial title and abstract screening for eligibility were conducted in unison. Following the initial screening at the title/abstract level, two researchers cross-checked 10% of exclusions against the inclusion criteria. The remaining publications that met the inclusion criteria were organised and allocated randomly to continue full-text screening for eligibility. In this phase, 100% of included and 25% of excluded papers were reviewed by a different reviewer (CV-P). Both screening at title and abstract and full-text level were performed by 4 reviewers (GA-G, TM, ND, FR). Detailed inclusion and exclusion criteria are depicted in (Table 1).

**Table 1:** Inclusion and exclusion criteria.

Inclusion	Exclusion
• Peer-reviewed articles or manuscripts where genomic programmes/services are mentioned and/or described. • Last five years. • No restrictions based on study location or language. • National or regional scale (when the scale was unclear, include those with multi-sited implementation). • Both diagnostic and screening purposes.	• Articles related to therapeutic genomic programmes. • PhD theses, dissertations, books, or conference proceedings. • Incomplete versions and articles where we could not access the full text.

### Data extraction

Data extraction was conducted using an extraction form on REDCap software to organise the review process. The extraction form was first piloted, and necessary amendments were made before extracting data from the included documents. Data were extracted by four reviewers and checked by a different team member.

### Data synthesis

Data were synthesised using framework analysis [13]. The analysis focused on developing themes that can accurately represent the data. The categories for the framework were based on the research questions guiding the review and the information emerging from the documents.

### Quality assessment

The methodological quality of the empirical articles was critically appraised in parallel to data synthesis using the Mixed Methods Appraisal Tool (MMAT) [14, 15]. The MMAT was developed to allow reviewers to assess the methodological quality of diverse study designs, including qualitative, quantitative, and mixed methods. Selected studies catalogued as reviews were not assessed using the MMAT since this tool is only indicated for quantitative, qualitative and mixed-methods studies. Overall, papers had an average score of 4.4 of 5.0. Lowest scores were related to insufficient interpretation based on the data and inconsistencies in using methods [16, 17]. The results from the assessments can be found in Appendix 2.

## Results

### Article selection

**Fig. 1:**
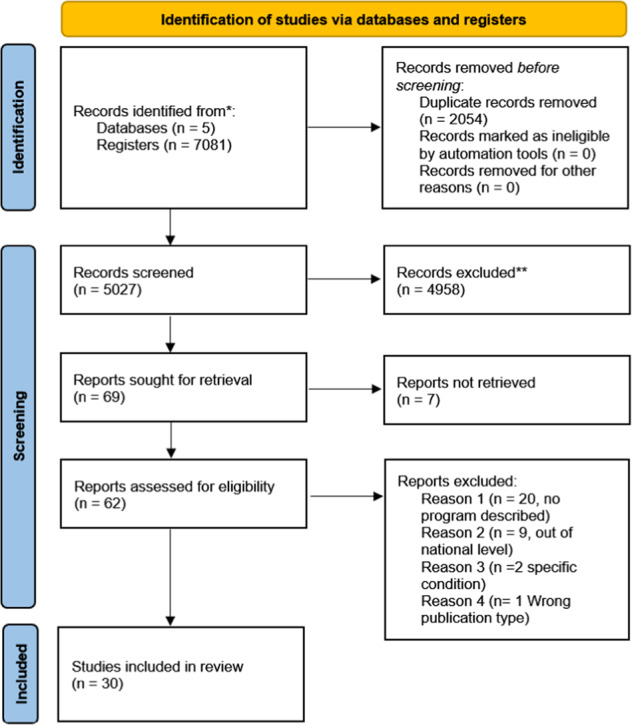
PRISMA flow chart (Preferred reporting items for systemic reviews and meta-analyses). Indicating the selection process of eligible studies.

The initial search yielded 5027 articles (after duplicates were removed). In total, 4958 articles were excluded as these did not meet the inclusion criteria outlined above. We reviewed 62 articles at the full-text level and excluded 32 because they did not describe genomic programmes, did not include implementation at a national or regional level or were focused on specific conditions. The final review included 30 articles (see Fig. 1 for the PRISMA Flow Diagram).

### Article characteristics

The main article characteristics are summarised in Table 2. Seven articles were from Australia, six from the US, four from France, three from the UK, one from Canada, one from the Netherlands, and eight were global in scope, including countries such as Pakistan, Estonia, Nigeria, Malaysia, Slovenia, Cyprus, Israel, Saudi Arabia, Belgium and Sweden. Sixteen articles described programmes implemented at the national level, four at the regional level, and eight described multi-sited programmes but did not specify if these were regional or national in scale. Fourteen articles focused on the use of genomics in screening, three focused on diagnostic genomic testing, and thirteen addressed both. Twenty-one articles were non-empirical reports in the form of commentaries, while the remaining nine articles were empirical in nature, of which seven were qualitative, one was quantitative, and one was mixed methods.

**Table 2:** Main article characteristics.

Surname/first author	Year of publication	Study location	Type of genomic testing programme	Type of article
Balasopoulou	2017	Malaysia	Screening & Diagnostics	Non-empirical report
Gaff	2017	Australia	Screening & Diagnostics	Non-empirical report
Sperber	2017	USA	Screening	Qualitative study
Spackman	2017	UK	Screening	Non-empirical report
Bertier	2018	France, Quebec	Screening & Diagnostics	Qualitative study
Laviolle	2018	France	Screening	Non-empirical report
Nadauld	2018	USA	Screening	Non-empirical report
Zebrowski	2018	USA	Screening	Qualitative study
Abimiku	2019	Nigeria	Screening	Non-empirical report
Delatycki	2019	Australia, Cyprus, Israel, Italy, Malaysia, Netherlands, Saudi Arabia, UK, USA	Screening	Non-empirical report
Burns	2019	Australia	Screening & Diagnostics	Non-empirical report
Levy	2019	USA	Screening	Qualitative study
Long	2019	Australia	Screening	Mixed-methods study
Pearce	2019	USA, UK, Canada, Netherlands, Estonia, South Korea, Cuba	Screening	Non-empirical report
Riaz	2019	Pakistan	Screening & Diagnostics	Non-empirical report
Rowe	2019	UK	Screening	Non-empirical report
Stark	2019	UK, France, Australia, USA	Screening & Diagnostics	Non-empirical report
Gaille	2020	UK, France	Screening	Non-empirical report
Snir	2020	USA	Screening & Diagnostics	Non-empirical report
Tonkin	2020	19 countries	Screening	Quantitative study
White	2020	High-income countries	Screening	Non-empirical report
Best	2021	USA, Netherlands, Australia, UK, Belgium, Sweden	Screening	Non-empirical report
Denommé-Pichon	2021	France	Screening	Non-empirical report
Elsink	2021	Netherlands	Diagnostics	Quantitative study
Long	2021	Australia	Screening	Mixed methods
Lynch	2021	Australia	Screening & Diagnostics	Quantitative study
Prins	2021	Estonia	Screening & Diagnostics	Non-empirical report
Traversi	2021	Italy	Screening	Non-empirical report
Vidgen	2021	Australia	Screening & Diagnostics	Non-empirical report
Vinkšel	2021	Slovenia	Diagnostics	Non-empirical report

### Implementation processes for programmes at a national scale

Most of the genomic programmes implemented at a national scale were in the pilot phase. The articles focussed on clinical settings [16, 18–25], governmental perspectives on genomic programmes [20, 21, 26–31], and, to a lesser extent, on the patients’ or participants’ experiences of these programmes [22, 27].

The cost-effectiveness and the funding of genomic programmes were recurrent concerns in implementing sustainable systems. It was frequently mentioned that the short-term funding of genomic programmes could hamper long-term continuity [26, 29, 32], and without palpable high-quality evidence, some governments could not justify creating ongoing publicly funded programmes [28]. Authors also frequently mentioned the need for an infrastructure capable of storing a large amount of confidential data and keeping up with the rapidly evolving technological landscape of genomics [16, 20, 21, 24, 27, 32–35]. Many articles also emphasised the importance of setting up an evaluation framework to obtain feedback and make changes [18, 27, 36–38].

Genetic counselling services were also regularly mentioned as a key means to supply information to patients, families, and the general public [22, 25, 27, 28, 32, 37, 39–41]. Some articles also highlighted that having transparent and standardised clinical pathways facilitated implementation [18, 24], but this needs to be accompanied by training programmes for healthcare providers to be effective [3, 20, 22, 25, 30, 37, 42–44]. Finally, topics such as culturally appropriate communication for patients and families, time efficiency in the delivery of services and interdisciplinarity of the staff involved were also mentioned as integral parts of programme implementation [9, 20, 21, 23, 25, 27, 32, 34, 35, 41]. Additional information can be found below in (Table 3).

**Table 3:** Main processes described in the articles.

Author/Year	Area of focus	Process of implementation	Description
Balasopoulou (2017)	Presents findings on the landscape of genomic testing and genetic counselling services in Malaysia.	Public and private genetic testing laboratories	This study identifies genomic laboratories providing genetic services in Malaysia where there is a high prevalence of genetic disorders such as hemoglobinopathies and metabolic disorders. • Need to establish databases that allow the storage and management of genetic and clinical data • Conferences to raise awareness • Genome-wide association studies • The University of Malaya is engaged in collaborative work with the Golden Helix Foundation to establish public health policies in the areas of pharmacogenomics and precision medicine.
Gaff (2017)	Implementation of a genomic programme across multiple autonomous institutions.	Collaborative, holistic approach for a phased implementation.	• Development of a proof-of-concept model including governance, policies, procedures, infrastructure, and software applications. • Development of pathway for patient testing. • Evaluation following hybrid effectiveness and implementation design.
Sperber (2017)	Challenges to genomic programme implementation in clinical settings, and strategies to overcome them.	The IGNITE (Implementing GeNomics In pracTicE) six projects vary in scope and design.	• Exploration of the use of genetic markers for disease risk prediction and prevention, development of tools for using family history data, incorporating pharmacogenomic data into clinical care, refining disease diagnosis using sequence-based mutation discovery, and creating novel educational approaches.
Spackman (2017)	Quantifying the value of genomic tests using Cost-Effectiveness Analysis (CEA).	Iterative approaches.	• The goal of the genomic test must be explicit. • Consider potential additional findings/multiple disorders.
Bertier (2018)	Usage, limitations and benefits of the clinical use of Next Generation Sequencing (NGS) for paediatric patients.	Focus on four teams, two in France and two in Quebec. Two teams used whole exome sequencing (WES) to improve the diagnosis and treatment of paediatric patients and families affected by rare diseases. The two other teams used it to help paediatric patients understand their absence of response to standard treatments and find more effective alternative treatments.	• Public institutions in both countries have invested significant funding in NGS following a political push for personalised medicine. • NGS is usually not considered to be routine care.
Laviolle (2018)	Recommendations to associate genomics with modern medicine based on reflections of scientific applications and operational and societal challenges.	Based on the “France genomics 2025” programme, which aims to integrate genomic tests into clinical practice for validated indications and develop a national genomics network including industrial partnerships.	• 4 pilot projects were set up in a diversity of contexts. • 13 working groups in charge of driving coordination, pilot projects, industrial participation management, ethical, regulatory, or medico-economic aspects, training, and communication. • 1 centre to define, validate, and implement the operational standards on sequencing platforms and data analysis and ensure the technological and computer research and developments required to deploy genomics.
Nadauld (2018)	Demonstrate the approaches and challenges associated with clinical implementation efforts designed to advance this treatment paradigm.	Precision oncology medicine programs are implemented by an integrated delivery system, a community care centre, and an academic medical centre.	• Pilot programme within a three-hospital region in the delivery network. • The program was subsequently expanded across all twenty-two hospitals and associated clinics. • The precision medicine workflow in oncology consists of three key features: an in-depth genomic analysis of the patient’s tumour, interpretation of genomic test results by a molecular tumour board (MTB), and drug procurement services.
Zebrowski (2019)	Implementation of genomic medicine in clinical settings (as opposed to individual level)	Based on IGNITE programme	• Apply the Consolidated Framework for Implementation Research (CFIR) to identify system-level factors that played a role in the implementation of genomic medicine within Implementing GeNomics in PracTicE (IGNITE) Network projects.
Abimiku (2019)	Build an internationally recognised biorepository for the receipt, processing, storage, and distribution of biospecimens for biomedical research. With a focus on the Institute of Human Virology Nigeria (IHVN) H3Africa Biorepository (I-HAB) in Abuja, Nigeria	Quality management system (QMS)	• Despite infrastructural challenges and limited resources, it is possible to establish a biorepository in a resource-limited setting that operates at an international level, if resources are leveraged to support the methodical implementation of a strategy for improvement that is grounded in established best practices and continuous monitoring and adjustment to local challenges.
Delatycki (2019)	To identify different approaches to reproductive carrier screening across a variety of different countries.	Different programs target distinct groups (high school, premarital, couples before conception, couples attending fertility clinics, and pregnant women) as does the governance structure (public health initiative and user pays). Ancestry‐based offers of screening are being replaced by expanded carrier screening panels with multiple genes that are independent of ancestry.	• Illustrates how the variability in how RCS is offered across the globe. This relates to geographical variation in carrier frequencies of genetic conditions and local health care, financial, cultural, and religious factors.
Burns (2019)	Examine critical considerations in successfully integrating genomic technologies into healthcare systems from a government perspective.	The priority areas for successful implementation are genomic services, data, workforce, finances, and person-centred care.	• Services: evaluation, ongoing advice, policy statements and national guidelines. • Data: Sufficient data-storage capacity, data sharing technology, governance around genomic data. • Workforce: Genomic education, interdisciplinary clinics, counselling and consent. • Finances: government investment in basic infrastructure and workforce and development of expertise. • Person-centred care: Culturally appropriate public health education and promotion programs.
Levy (2019)	To describe findings from the National Human Genome Research Institute’s (NHGRI) IGNITE Network in identifying key constructs, opportunities, and challenges associated with driving sustainability of genomic medicine in clinical practice.	The primary driver–stakeholder dyads were: • Genomic training for providers, genomic clinical decision support (CDS) tools embedded in the electronic health record (EHR) third-party reimbursement for genomic testing.	• Six research institutions and 14 community partners funded to demonstrate the feasibility of genomic medicine in diverse settings. A further 16 affiliate institutions voluntarily collaborate with IGNITE to learn genomic medicine implementation techniques, share their experiences, and participate in network activities
Long (2019)	This study aimed to map and analyse interconnections between the members-a key feature of complexity-to capture the collaborations among the genomic community, document learning, assess Australian Genomics’ influence and identify key players.	A collaborative approach to implementation based on ‘hands-on learning’ and ‘making group decisions’	• Successful implementation of genomics requires the engagement of multidisciplinary teams across a range of conditions - Australian Genomics is facilitating this collaborative process by strategically building a genomic learning community
Pearce (2019)	Assess the readiness of the United Kingdom (UK) National Health Service to implement a Genomic Medicine Service	Mainstreaming	• The organisational, social, and cultural implications of reforming practice, highlight that demonstration of clinical utility and cost-effectiveness, attending to the compatibility of genomic medicine with clinical principles, and involving and engaging patients are key to successful implementation.
Riaz (2019)	Focus on Pakistan and the challenges to implementing genomics in the national healthcare system -Preconception carrier screening or pre-natal -Screening for chromosomal abnormalities screening programme - the Congenital Hypothyroidism Screening Programme	Propose an achievable, staged approach for the implementation of PHG, which includes setting short-term (3–5 year) goals, followed by longer-term (10–15 year) goals.	• Pakistan still lacks a national newborn screening programme, clinical genetic testing services, or public health genomics framework, despite having the world’s highest rates of inter-family marriages and prevalence of inherited genetic conditions ○ Initial steps towards strategic prioritisation, resourcing, and long-term goal setting are required.
Rowe (2019)	Evaluate the Expanded Universal Carrier Screening (EUCS) programmes.	There are considerable differences in panel composition between laboratories.	• The primary objective of a programme should be increased reproductive autonomy. • Efficacy should be assessed by how the programme optimises informed choice. • When and where EUCS could occur needs careful consideration, as it will influence cost, uptake, and the reproductive options available to couples.
Stark (2019)	To review the diverse approaches and current progress made by national genomic-medicine initiatives in the UK, France, Australia, and the US and provide a roadmap for sharing strategies, standards, and data internationally to accelerate implementation.	Evidence-based implementation through collaboration and data sharing	• These national genomic-medicine initiatives are driving transformative change under real-life conditions while simultaneously addressing barriers to implementation and gathering evidence for wider adoption.
Gaille (2020)	Presents a joint position of the UK-France Genomics and Ethics Network (UK-FR GENE), which has been established to reflect on ethical and social issues arising from the integration of genomics into routine clinical care in the UK and France.	National programmes funded by the state	• Despite each country’s strategy being at a different stage of implementation, the two countries face similar ethical issues • each country tries to solve these issues by (re-)defining individual rights and collective duties in its way • the social contract presents a useful tool to analyse the ways the UK and France address the ethical challenges raised by genomics.
Snir (2020)	Implementation challenges in the scaling of clinical genomic services.	Integration of software to support specialists such as genetic counsellors and medical geneticists in integrating genetics into primary care.	• Development of software to assist in electronic health record integration, the education of patients and providers, tools to stay abreast of guidelines, and simplification of the test ordering process. • Proposes online educational videos, telehealth services, software to facilitate family history record taking, chatbots to answer frequent questions and conduct preliminary triage.
Tonkin (2020)	Guiding nurse leadership in the integration of genomic programmes in their services.	Proposes a maturity matrix to support the implementation of genomic programmes by nurses.	• Exploring and scoping factors of potential influence for change. • Readiness planning (baseline/needs analysis, funding, governance). • Raising awareness with stakeholders; building capacity and capability; mobilising resources. • Active commitment to engage with and implement change. • Culture of ongoing improvement in genomics is embedded, valued and sustainable.
White (2020)	To identify the barriers and facilitators to integrating genetics and genomics into nurses’ and physicians’ usual practice	Mainstreaming	• Building the capacity of nurses and physicians to integrate genetics and genomics into routine clinical care is essential if opportunities afforded by precision medicine are to be fully realised.
Best (2021)	Focuses exclusively on healthcare practitioners’ perceptions of barriers and enablers of reproductive genetic carrier screenings (RGCS) - because literature tends to focus on the attitudes of patients and families of those affected by genetic conditions.	Identifying the determinants of implementation is an essential first step in designing implementation strategies to overcome barriers.	• The use and potential impact of RGCS, including factors influencing equity of service take up and focus on the client • Practitioners’ beliefs and expectations about the process of delivering RGCS, including the ability to deliver RGCS, knowledge about and support for RGCS, opinions about RGCS, and external influences on practitioners • Resources available for practitioners for RGCS, including counselling, models of care delivery and other nonclinical barriers to delivery of RGCS.
Denommé-Pichon (2021)	FASTGENOMICS is a French national, multicentre, prospective pilot study in new-borns and infants suspected of genetic disease and hospitalised in neonatal or paediatric ICUs.	The pilot is designed to evaluate the feasibility of implementing trio-GS to deliver a result in less than 45 days while minimising extra costs by adapting the organisation to integrate urgent requests as a priority into the usual, not urgent workflow to identify the technical or organisational obstacles encountered.	• Speeding up the time to diagnosis using GS. • Includes patients from multiple hospitals but relies on only one laboratory and one sequencing platform. • Limit additional costs.
Elsink (2021)	Inborn errors of immunity (IEI) are a heterogeneous group of disorders, affecting different components of the immune system. This makes the early application of next-generation sequencing (NGS) as a diagnostic method in the evaluation of IEI a promising development	Early application of an NGS-based IEI panel	• With the rapidly evolving field of IEI-related genes, assessing genetic defects within these patients should be an ongoing process; periodic reanalysis of the WES data is advisable
Long (2021)	To develop a rich picture of the entire national Australia genomic program and its link and relationships with the broader context and show key stakeholders, agencies and processes and their interdependencies. (Holistic study of the programme)	Six research institutions and 14 community partners funded to demonstrate the feasibility of genomic medicine in diverse settings. A further 16 affiliate institutions voluntarily collaborate with IGNITE to learn genomic medicine implementation techniques, share their experiences, and participate in network activities.	• Uncertainty: who owns the data? How will it be reused in the future? How to deal with unexpected findings? will everyone adapt and adopt genomics modern technologies? what is the demand for genome sequencing? • Non-linear processes: i.e., patients worry about how genomic tests may affect their insurance • Unintended consequences: time commitments and need for more funding • Interdependencies: nature of the workforce is linked to funding, which is linked to capacity and experience.
Lynch (2021)	To explore parents’ experiences of rapid Genomic Sequencing (rGS) for their critically unwell infant or child	Feasibility programme	• Identifies tensions between the medical imperative of rGS and parents’ decision-making, which need to be addressed as rGS becomes routine clinical care.
Prins (2021)	The way advances in genomic research will transform the future of personalised prevention and medicine in Estonia.	Translational research	• Genetic discoveries are improving personalised prediction and advance functional insights into the link between genetics and disease
Traversi (2021)	Review the emerging field of public health genomics in Italy and its integration into sanitary regulations and governance instruments.	Since 2013, personalised health has been a pillar of the National Prevention Plan. Recent educational efforts geared towards professionals, citizens, and decision-makers complement it.	• Genomic predictive tests are used in public settings to investigate monogenetic disorders. • Genetic screening for complex diseases has been applied to a few conditions. • Since 2011, two practical distance training courses on genetics and genomics have been released for physicians. • Other initiatives were directed at a larger audience of healthcare professionals.
Vidgen (2021)	Presenting the genomic program in Queensland and the model for its integration into the broader healthcare system.	The structure and management of the Queensland Genomics program are based on adaptive management philosophy.	• The adaptive management strategy establishes processes that feedback lessons learnt based on experiences of running the program and have mechanisms to enable change reflecting this learning. • It is applied in complex adaptive systems, such as healthcare so that programs can operate in situations of uncertainty. • This program model was selected as genomics is a discipline experiencing fast technological changes and an evolving knowledge base.
Vinkšel (2021)	Implementation of NGS in diagnosing rare diseases and present advantages and challenges of diagnostic approach, with a focus on Slovenia.	NGS testing is offered via a clinical genetic service.	• Ensures responsible and efficient use of the recent technology to achieve economic sustainability. • Patients referred from various medical specialities are evaluated by a clinical geneticist who checks for appropriateness of the referral (diagnostic hypothesis, probability of genetic aetiology, clinical utility), performs phenotyping of the patient, communicates with the NGS diagnostic unit in terms of interpretation of the result and provides pre and post-test genetic counselling.

### Implementation barriers and enablers

#### Factors acting as barriers to implementation

Various barriers to implementing genomic programmes were mentioned across the 30 articles, many of which were interrelated. Nineteen articles identified the lack of genomic education and literacy and the need for significant upskilling of the healthcare workforce as a barrier. Fourteen articles identified the significant costs of enacting system-wide change as a barrier to implementation, with many pointing to the costs involved, not only for running tests and analysing them, but also the additional time and staff needed to provide adequate education, training, consent, and counselling, and the costs involved in integrating genomics information and technology into the healthcare system. Nine articles also perceived a lack of guidelines, regulations, and standards as a significant barrier, which may be related to the perceived lack of government and policy-making support for genomics. Six articles identified the lack of integration between Electronic Health Records (EHR) and genomics data as a barrier to successful implementation, particularly considering healthcare fragmentation and the need for EHRs to be accessible to patients to share across different healthcare organisations [22, 35]. Two articles identified that genomic medicine implementation is hindered by the quantity and types of evidence required for healthcare payers to justify reimbursement, being unwilling to reimburse for new diagnostics that do not meet their thresholds for clinical validity and utility [37, 45]. One article also identified parents’ emotional, psychological and time costs as a barrier to rapid genomic sequencing in intensive care, compounded by counselling in stressful hospital intensive care settings [41].

#### Factors acting as enablers in implementation

Ten articles discussed the need to incorporate genomics into medical and nursing education programmes and provide training to existing healthcare workers in conjunction with enhanced regulation, guidelines, and standards [44]. Ten articles identified a combination of the increasing accessibility and affordability of genomic technology (e.g., rGS and NGS) and increasing data storage capacities as factors facilitating the implementation of genomic programmes. Eight articles highlighted the value of lesson-sharing and collaborative communication between hospitals, clinicians and countries. Four articles discussed the need for health services to accommodate genomics at an organisational level, including integrating genomic data and EHRs and embedding programmes into clinical portfolios, such as cancer and infectious diseases [24]. Eight articles discussed the role of genetic counselling, the use of decision aids, patient engagement and education in facilitating implementation. Finally, two articles discussed the need for modelling to demonstrate implementation at a larger scale, with a focus on integration and employing adaptive management philosophies that allow for flexibility to facilitate changes in response to learning and external factors [18, 24]. Further details about the main barriers and enablers discussed in the articles can be found in Appendix 3.

## Discussion

This rapid evidence review synthesized implementation barriers and enablers across several countries and identified challenges and opportunities to consider for implementing genomics diagnostic or screening programmes. As such, this section will discuss some key areas and themes from the findings. It is important to bear in mind that these enablers and barriers to implementation vary across geographical areas and countries. As such, they should be considered general barriers and enablers that will affect different countries and areas differently. It is also important to note that addressing any one of these factors would not be sufficient to guarantee implementation; a holistic approach must be taken to address micro and macro-level implementation challenges.

### Genomic education and training of healthcare staff

In line with the rapid advancement of genomics across the fields of medicine, science, technology, ethics and legislation, there is a need for continuous education and the exchange of knowledge between different stakeholders to keep up with the pace of progress [46]. The successful integration of genomics into healthcare fundamentally depends on healthcare professionals with the appropriate genomic knowledge and skills, and this was the most cited factor in the articles in this review. For the promise of genomic medicine to be translated into improved patient outcomes, it is necessary to establish quality evidence-based education with clear objectives for learning [47]. This would require integrating genomics into training for the upcoming workforce (e.g., though undergraduate, graduate, or specialist professional training), as well as initiatives to target the existing health care workforce who would not have had any prior education in genomics or who need to refresh their education to reflect more recent developments. For these interventions to be successful, there is a need for reporting standards which entail consistent descriptions enabling replication, comparisons and helping those developing interventions to learn lessons from past efforts. An example of this is a program logic model and reporting standards for genomics education and evaluation, proposed following international collaboration and consensus [47, 48]. Of note, there is no overarching governing body to publish or mandate the use of reporting standards for education interventions for genomics, but global and national bodies can facilitate awareness and implementation.

It is also essential to develop online learning tools that improve accessibility internationally and facilitate sharing educational efforts [27]. Most healthcare professionals prefer education that demonstrates clinical utility via workshops, lectures, conferences, or online materials [44]. Indeed, it is noted that the motivation to learn new skills is tied to the applicability of the information [49].

Other approaches include developing cross-professional genomics competency frameworks in [50–52]; utilizing techniques such as the ‘flipped classroom’ blended learning approach [49]; and embedding trained genomic specialists within mainstream clinical settings to provide on-hand education and support [16]. These strategies can provide greater consistency, encourage multidisciplinary team working, and lead to more effective practice through more flexible and constructive use of healthcare professionals’ time, and focusing in-person interactions on case-based learning or interactive discussion [17, 49–52]. Hands-on learning or experiential learning approaches are recognized ways to support the development of skills and confidence in genomics [17, 52]. Clinical Decision Support (CDS) tools can also help clinicians with limited knowledge by providing just-in-time information with links to evidence [53].

While education is not the sole factor guaranteeing the success of genomic programmes, it is clearly integral to provide a foundation for implementation. Crellin et al., (2019) highlight that adults are most likely to be receptive to educational interventions when they recognize a need to learn, and the benefits of the knowledge acquired. Many specialists continue to hold concerns about genomics including a lack of clinical guidelines, the often-uncertain nature of genomic results, and the potential to cause psychological harm to patients; nevertheless, most acknowledge that integrating genomics into healthcare is inevitable [54]. It is important to ensure that education is not addressed in isolation, but considered in the context of other factors such as the ability to enable multidisciplinary team working and coordinated care pathways, patient and public perspectives, and ethical issues surrounding genomics.

#### Public support and patient education and engagement

The successful implementation of genomics will also depend on the culturally appropriate engagement of the general public, for which public health education and genomics promotion programs are necessary [27, 53]. Such programmes should draw on behavioural economics and deliberative public engagement methods [27]. Common approaches to engaging the general public include educational events, online platforms, mass media and social media engagement [16, 46]. In Finland, the government plans to educate its citizens on making informed decisions regarding genetic testing and study participation. It intends to implement guidelines on using genomic data, such as the educational genome portal and virtual health services, to enable people to engage with and learn to use their genomic data [46]. The UK, France, Canada, Denmark and Finland have all started integrating genomic education into the primary and secondary education curricula, using online platforms for education, holding educational symposiums and providing specialised training to teachers [46, 55]. In New England, the professional development programme Teaching the Genome Generation (TtGG) provides teachers with the necessary tools to educate students on genomics through biology classes [56].

Patient engagement and education is crucial as it is the stakeholder group most impacted by genomics, and strategies to address this must go hand-in-hand with approaches to educating healthcare professionals as discussed above. This can be provided via workshops, community outreach programmes, interactive and multimedia e-learning tools and decision aids to support consent [22, 46, 57, 58]. Actively involving diverse groups of patients in implementation, for example through patient advisory boards or through co-design of information materials, has also shown to enhance success of implementation [16].

#### Communication and counselling

Communication between various stakeholders is crucial to developing sustainable genomic programmes. For instance, interactions between and within teams in hospital settings are essential to long-term success [27, 35, 42]. Tools such as webinars, conference calls, and presentations between the teams involved in the programmes encourage collaboration and enable feedback and eventual modifications while also unifying the different strands of the project [17, 35]. Team members of the American-based IGNITE projects thought this strategy was critical to engagement and buy-in by physicians and hospital management – which ultimately facilitated the incorporation of genetic medicine into routine care [35]. For Australian Genomics, a study measuring collective ties between clinicians showed that “hands-on learning” and “making group decisions” were the most potent influences on their genomic practice. This was achieved by strategically building a genomic learning community by creating boundary-spanning roles [17].

Although genetic counselling is considered best practice, several barriers exist to its successful implementation. Genetic counsellors are a highly specialized resource with limited numbers, and therefore difficult to scale. Models to facilitate large-scale delivery could include mainstreaming genetic counselling skills among non-genetic experts (such as nurses, pharmacists and general practitioners), or using digital tools such as telehealth or chatbots to help triage patients and support decision making [22, 27, 59].

Challenges also exist with regards to patient communication. In some circumstances, such as critical care settings, patients are required to understand information quickly in high-stress environments [41]. Information must therefore be sufficiently tailored to support patents’ diverse decision-making needs. Social factors such as discouragement by family members, health insurance coverage, ability to pay, and lack of awareness of available genetic services from patients and clinicians have also been identified as barriers to accessing genetic counselling [22, 59]. As well as public, patient and healthcare professional education, successful strategies to overcome these barriers include delivering genetic counselling in more accessible locations (such as primary care facilities) or providing this service remotely [32]. Genomic counselling seeks to help patients “understand and adapt to the medical, psychological and familial implications of genetic contributions to disease” [57] and is an important facilitator to information about genomic technologies.

Advocacy actions and information campaigns improve public awareness of genetic technologies. They are likely to make access to genomics information easier for clinicians and patients and, in turn, facilitate the implementation of genomics into routine care [20]. Nevertheless, these public interventions must be culturally appropriate and accessible to be effective [24, 27]. However, there is a gap in the literature regarding the public perception of genomic technologies, their needs and expectations, and their experience with these techniques. This sociological information would help all stakeholders by helping orient research perspectives and improving the guidance and support offered to patients.

#### Regulatory frameworks

As the field of genomics progresses rapidly, there remains the problem of insufficient clinical evidence combined with a lack of ethical, legal and social regulations [30]. Indeed, fully realising genomic medicine’s potential requires a multi-pronged research, clinical, policy and regulatory agendas. As genomics evolves, so does the regulatory landscape around it, albeit in an often uncertain and ad hoc manner. As well, there are limitations to understanding genetic links between health and disease, particularly for rare conditions, when data is scarce or siloed. For its potential to be truly harnessed, genomic research and healthcare data need to be integrated and accessible at a large scale [60].

Regulatory approaches to genomics in research, clinical care and public health vary with regards to issues of liability, consent, quality assurance, and data and privacy protection. Increased availability of direct-to-consumer (DTC) testing creates additional challenges as this is accessed outside of health care and research initiatives and is typically unregulated [60, 61]. The current approach to research, clinical care, public health, and DTC testing is siloed and fails to recognise how genomics data is produced and used across these areas [61]. Policymakers and legislators need to regulate the return of results, confidentiality and privacy while creating policies that promote genomic education and economic incentives that bring together the interests of different stakeholders [62]. The convoluted ecosystem of stakeholders necessitates technical standards that are neutral and adaptable for diverse purposes and relevant to the wide-ranging set of clinical, research, commercial and public users [63].

It is widely acknowledged that the potential of genomics-enabled research and clinical care is proportional to the amount of data that can be accessed and analysed and the implications shared [62]. As such, regulatory frameworks for genomics need to be perceptive of the connections blurring the boundaries between research and clinical care, such as when sequencing activity should be considered research or care and how findings with clinical implications should be managed and by whom [33]. Pooling data can also help increase sample sizes to a level that makes investigating every rare condition feasible. In 2018, 21 European Union (EU) member states signed a joint declaration to enable the cross-border sharing of human genomes by 2022 [60]. However, genomic data may allow the re-identification of donors, which has privacy implications and is also sensitive regarding fundamental rights. The 2016 General Data Protection Regulation (GDPR) establishes the rules for personal data protection and sharing by individuals, companies, and organisations at the EU level. However, the reality has not lived up to hopes, with the member states using their discretion to interpret and implement the regulation differently [64]. Challenges also exist within countries, where health care is administered at a provincial or state level; for example, differences in legislation and common law interpretations between Australian states create challenges for researchers and clinicians, leading to uncertain expectations of how institutions will protect data [65]. Shared guidelines or codes of conduct effectively provide practical guidance and procedural clarity to avoid inconsistencies. Researchers and scientists can participate in their development, thereby increasing the chances that data protection will be addressed in line with the relevant sector and reinforcing the code’s factual and scientific legitimacy [64].

Ultimately, codes of conduct ease responsibility issues by clarifying the appropriate safeguards for genomic data protection. They promote the coordinated application of rules and, over time, they directly influence the laws governing data sharing by enabling improved engagement with data protection principles and an enhanced understanding of obligations [64].

Differences in legislation and common law interpretations between Australian states create challenges for researchers and clinicians, leading to uncertain expectations of how institutions will protect data [65]. Creating more precise expectations and encouraging public trust would be better promoted by having a more harmonised regulatory approach. Public trust is vital to collecting, storing, and sharing genomic data [66]. Regulations promoting accountable and transparent collaboration and standardised data collection and reporting are critical to fostering such trust.

A federated approach is seen as a viable strategy in cases where data cannot be pooled for legal or practical reasons [60]. Under this approach, independent bodies host the data in a secure processing environment governed by technical standards that enable large-scale analysis [63].

Although challenging, international collaboration is crucial to addressing regulatory issues relating to genomics in research and health care, and maximising the value of data. Consortia such as the Global Alliance for Genomics and Health (GA4GH), including researchers, data scientists, healthcare professionals and patient advocacy groups, are working towards consistent standards in regulation, policy and data management to enable more effective comparison across countries [62, 63]. Even at the level of electronic patient health records, EHR software vendors are highly proprietary, creating complications for standardisation and harmonisation with research systems [63]. Standard methods for collecting, storing, sharing, accessing and analysing genomics data need to be agreed upon if the benefits of genomics medicine are to benefit the population at large.

Legal differences across domains should be harmonised to reduce confusion and increase compliance. For instance, regulatory differences between research and clinical care cause unnecessary confusion concerning data identifiability, deidentification, and re-identification [61]. There is a need for stakeholders to have clarity and education about regulations governing the different domains of genomics to ensure transparency and accountability.

In the absence of widely accessible genomics services via national healthcare systems, DTC companies have generated a market and grown rapidly, leading to decreased costs and increased access to genetic tests [67]. However, governments today remain unable to monitor or quantify DTC testing effectively and are limited in their ability to regulate it [27]. DTC tests are often delivered without oversight from a healthcare practitioner, results may be inaccurate or misleading, and some operate outside of regulatory frameworks entirely if they are based offshore and deliver services via the internet [68]. DTC testing may be addressing a gap in public interest and need in the absence of sufficient access to genomic testing in health care, but risks causing greater complications with regards to the barriers mentioned in this review including regulation and public trust [68, 69]. As such, policymakers need to develop to regulation and medical coverage approaches that enables genomics tests to be the most effective pathway to clinical care [62].

#### Ethical considerations

There are a number of ethical issues raised by genomics in research and health care which can significantly influence implementation of genomics programmes. Should the genomic test be regarded as research or as clinical care? What are the responsibilities of the clinician and/or researcher to feedback research findings to participants? Should relevant results be communicated to family members as well? How can genomic programmes be designed and delivered in an equitable manner? The answers to these questions have variable implications for implementation of genomic programmes, including workforce capacity; regulatory frameworks;as well as public and patient engagement and trust.

It is generally acknowledged that patients should be kept informed about the type of information they may receive throughout the genomic testing process (whether clinical or research-based), and be respectful of their choices regarding the types of results they wish to receive. There are recognized challenges to facilitating valid consent while addressing the fact that genomic results can be complex and uncertain [33]. To enable this, patients must be empowered to actively participate in decisions about their care, which includes having awareness of the ethical, legal and social implications of genomics [46].

### Recommendations summary

In discussing the barriers and enablers to the implementation of genomics programmes many of the articles reviewed also highlighted recommendations for moving forward which are summarised here.

It is recommended that genomic education needs to be improved and made more accessible for all stakeholders including the public, patients, healthcare providers, policymakers and governments [17, 27, 37, 44, 46, 49, 53, 54]. For such education interventions to be successful reporting standards comprising consistent descriptions which enable replication, comparisons and lessons learned are advocated [47, 48].

With regard to communication, it is recommended to establish genomic counselling services with communication and information tool guidelines to maintain good communication with patients and their families and enable informed decision-making [22, 27, 32, 33, 41, 46, 59]. It is also key to involve patients, participants and the public in decision making processes, and to incorporate their experience of implementations in it as well. Moreover, multidisciplinary engagement and communication across a range of conditions and expertise is advised [17, 18, 37, 43, 70].

Finally, to enable the successful implementation of genomics in healthcare articles advise developing appropriate policies and regulatory frameworks to govern genomics, with a particular focus on harmonising regulation across the domains of research and clinical care and establishing legislation regulating the collection, storage, and sharing of personal genomic data [33, 60–64].

### Limitation and strengths

The review used a rapid design, meaning that only a limited number of databases and websites were accessed, which facilitated timely results. It is possible that specific subject headings, keyword terms and synonyms have been missed. The review was strengthened by having a multidisciplinary team with five reviewers searching for peer-reviewed articles and cross-checking their relevance, following robust systematic research guidelines.

## Conclusion

This review has outlined the implementation process for genomic screening or diagnostic programmes on a national scale and the interrelated factors that act as barriers and facilitators to their implementation. It is essential to acknowledge the differences in policy, funding, etc within and between countries, and the extent to which they are affected by those barriers or enablers. Across the articles reviewed, most of the programmes were still in pilot phases and early stages. Genomic healthcare has grown over the past years, but a range of interlinked factors must be addressed for these programmes to thrive and translate into national-scale implementation. As is often the case, investment in research is not yet matched by clear financial and policy commitments to widespread implementation and workforce education, with many programmes only operating on a small scale with limited accessibility to the public and within a complex and convoluted international landscape. Taken together these findings can help to inform the design of future programmes, as well as future research on the factors influencing the implementation of population-based genomic programmes. Hopefully, the implementation of the programmes and initiatives discussed above will lead to durable national programmes and enable increased and equitable access to genomics in our healthcare systems.

## Supplementary information

Supplementary information
