# Supplementary material for: The implementation of large-scale genomic screening or diagnostic programmes: A rapid evidence review
Source: Eur J Hum Genet. 2022 Dec 14;31(3):282–95. doi: 10.1038/s41431-022-01259-8 (PMC9995480; doi:10.1038/s41431-022-01259-8)
Supplement: Supplementary file 1 — Supplementary information [file 41431_2022_1259_MOESM1_ESM.pdf]

Appendixes

Appendix 1. Search strategy

Themes

#1 GENOMICS

Structural genomics OR comparative genomics OR functional genomics OR mutation OR epigenomics OR nutrigenomics OR metagenomics OR proteomics OR proteogenomics OR imaging genomics OR radiation OR human genome project OR HapMap project OR cognitive genomics OR pharmacogenomics OR pangenomics OR genomic sequencing OR genomic medicine OR genotype OR genome.

#2 IMPLEMENTATION

Implementation science OR implementation mapping OR implementation research OR implementation strategy OR rapid implementation OR clinical implementation

#3 HEALTHCARE

Biomedical technology OR preventative health services OR health services research OR delivery of healthcare OR precision medicine OR precision health care OR evidence-based healthcare OR integrated healthcare OR medical care OR healthcare reform OR healthcare planning OR healthcare system OR healthcare innovation OR healthcare policy

Strategy

(structural genomics OR comparative genomics OR functional genomics OR mutation OR epigenomics OR nutrigenomics OR metagenomics OR proteomics OR proteogenomics OR imaging genomics OR radiation OR human genome project OR HapMap project OR cognitive genomics OR pharmacogenomics OR pangenomics OR genomic sequencing OR genomic medicine OR genotype OR genome) AND (implementation science OR implementation mapping OR implementation research OR implementation strategy OR rapid implementation OR clinical implementation) AND (biomedical technology OR preventative health services OR health services research OR delivery of healthcare OR precision medicine OR precision health care OR evidence-based healthcare OR integrated healthcare OR medical care OR healthcare reform OR healthcare planning OR healthcare system OR healthcare innovation OR healthcare policy).

Appendix 2. MMAT scores

Article	MMAT score
Sperber et al., 2017	***
Bertier et al., 2018	*****
Zebrowski et al., 2018	*****
Levy et al., 2019	*****
Long et al., 2019	***
Tonkin et al., 2020	****
Elsink et al., 2021	*****
Long et al., 2021	*****
Lynch et al., 2021	*****

The total possible score for each article is *****

Appendix 3. The main barriers and enablers discussed in the articles.

Theme and References	Barriers	Enablers
**<u>Genomic Education and Training of Healthcare Staff</u>**<br><br>(3,6,24,26,29,31,36,38,39,41–43,16,45,46,17–23)	• Overall lack of genomic education and training initiatives and resources available.<br>• Outdated physician practice patterns, including fee-for-service models impacting the ability to access training.<br>• Healthcare professionals have a non-accurate perception of liability and professional obligation when it comes to genomic testing.<br>• Access to genetic specialists is limited and insufficient Healthcare professionals lack knowledge, skills, experience and confidence relating to genomics (e.g., understanding how to request genomic tests, undertake an individual risk assessment, and interpret genetic variants and results.)<br>• Healthcare professionals' lack of knowledge and awareness blocks patients from accessing genomic testing, assessment of genetic risk, and/or being referred to genetic services such as genetic counselling.<br>• Genomic programmes require significant education and	• Development of educational material and training courses for clinicians.<br>• Creation of educational outreach with clinicians.<br>• Incorporate training and continuing education in genomics in medical school curricula.<br>• Engagement of multidisciplinary teams across a range of conditions and expertise to insure successful implementation.<br>• Clinician preparedness education interventions that are shown to improve genetics knowledge among clinicians.<br>• Expand genomic-focused Career Development Awards or Institutional Training Grants.<br>• Build on clinicians' existing understanding of the complexities of genomic sequencing.<br>• Ensure that systems and processes implemented are embraced by those intended to use them.<br>• Focus on experiential learning.

	upskilling in their initial phase as genetic awareness and literacy need to be established.<br>• Lack of awareness and skills leads to misconceptions about genomics (e.g., perceptions of eugenics and uses of data).	
**Public Support**<br><br>(3,26,27,32,46)	• Lack of public interest due to a lack of public understanding of genomics.<br>• Limited availability and access to education about genomics.<br>• Cultural and religious factors, such as differing attitudes and perceptions towards disability and reproductive choices.<br>• Screening could potentially be perceived as taking a discriminatory view of those already living with specific conditions, particularly where effective treatments exist.	• Culturally appropriate public health education and promotion programs.<br>• Deliberative public engagement methods.<br>• Partnership model between public healthcare systems and private companies to foster public trust.
**Patient Perspectives**<br><br>(3,20,21,38,42,44)	• Patients may experience worry or anxiety about genomic results; particularly those that create uncertainty.<br>• Patients may feel coerced into having tested at a particularly sensitive time (e.g., when seeking a diagnosis).<br>• Patients' lack of understanding of genomic testing and the implication of results can lead to misconceptions about the value of genomics.<br>• Patients seeking care within a disjointed healthcare system may not be aware of how their results are relevant to other aspects of their health or other family members.	• Online educational videos to promote patient awareness and uptake of genetic counselling and testing.<br>• Developing tools to support patients with informed consent (e.g., decision aids).<br>• Involve patients in implementation activities.<br>• Active engagement with patient advocacy groups.<br>• Activities to increase the knowledge of the general public, which would address current and future patients.
**Genetic counselling**<br><br>(6,24,26,38–40)	• Lack of formal recognition of genetic counsellors' role.<br>• Lack of legal requirements for the provision of genetic counselling services.<br>• Access to genetic counselling and offers of reproductive options vary significantly within and between countries.<br>• Challenge to find appropriate space/time for counselling in mainstream healthcare settings (e.g., intensive care units).	• Supportive and guided approaches to facilitate parents' decision-making, including individualised pre-test counselling where appropriate (e.g., rapid genome sequencing in intensive care).<br>• Digital tools (e.g., chatbots) to support the provision of information and triage patient queries - online delivery of information is equivalent to or non-inferior to in-person, group or telehealth counselling.<br>• Consistent advice and standards from professional bodies, to clarify healthcare practitioners' perceptions

		about liability and professional obligations.
**Policy and regulatory frameworks**<br><br>(6,16,26–29,31–33,36,38,39,17,41,44–47,18–20,22–25)	• Lack of standards, regulations and clear organisational policies<br>• Lack of leadership and agreed guidelines or standards for training.<br>• Lack of focus at an organisational/policy level on the relevance of genomics to public health.<br>• Insufficient support among policy makers and government.<br>• Challenge for policy development to keep up with the pace of change in the uses of genomic testing.<br>• Lack of consideration of the personal utility that genomic testing can bring, beyond clinical utility (e.g., broader benefits of a diagnosis, to inform future and reproductive planning)<br>• Lack of ethical frameworks to address issues, e.g., when genomic testing should be regarded as research vs clinical care, how clinically relevant findings generated during genomic research should be managed.<br><br>Difficulties in gathering sufficient translational evidence to inform policy, particularly for tests or conditions that only impact a small number of patients.	• Stronger leadership in transforming healthcare through policy development.<br>• Appropriate policies, regulatory frameworks and common principles.<br>• Clear criteria for quantitative and qualitative evidence requirements from payers and decision-makers, so that researchers know where to target their efforts.<br>• Creation of genomic health services external to public health organisations (e.g., pre-conception counselling clinics) to support access needs.<br>• Developing the technology internally as a cheaper solution to ordering genomic testing from elsewhere, and to build in-house expertise. Appropriate governance around the collection, storage, and sharing of national genomic data.<br>• Costs of implementation also encompass running the test and the extra time and staff needed to provide adequate education, consent, and counselling.<br>• Demonstration models enable the testing of implementation approaches.<br>• Implementation of feedback loops.
**Funding**<br><br>(3,27,31,39,41,45,46)	• High cost of genomic programmes, including testing, analysis and wider infrastructure costs.<br>• Unclear and inconsistent reimbursement policies for genomic tests.<br>• In many countries, funded testing is minimal or only available to those with a high a priori risk.<br>• Patients cannot pay and have insufficient health insurance coverage.<br>• Increasing population size with a	• Increase funding for education and training programmes.<br>• Enabling greater access to genomic testing.<br>• Support from governmental bodies: investment in infrastructure and workforce, embedding genomic expertise in national health services, and providing ongoing and more equitable funding.

	limited public health budget with competing priorities<br>• Lack of funding both within (i.e., rural/urban divide) and between countries	
**Infrastructure**<br><br>(3,16,31,34–36,41,42,46,18,19,21,23,24,26–28)	• Time pressures on healthcare workers.<br>• Prioritisation of other public health concerns, such as high infectious disease rates and hospital emergency services.<br>• Lack of genomic-focused clinical decision support (CDS) tools, and optimised clinical workflows.<br>• Limited access to genomic testing and indicated therapies (geographic access disparities as well as insurance compensation).<br>• Lack of coordination between genetics and non-genetic services, inequity and variability in provision.<br>• The lack of risk assessments by primary care physicians creates a barrier to accessing genomic testing and specialist care.<br>• Complex processes to obtain consent for genomic testing, whilst ensuring informed decision making.	• Increase accessibility of NGS technologies: increase accessibility and speed of diagnosis of genetic disorders.<br>• Early engagement of hospital leadership and management staff to ensure success and sustainability.<br>• Clinical decision support tools<br>• Agreement on types of genomic tests that are suitable for mainstream settings versus those that should be escalated to specialist genetics services.<br>• Reorganisation of diagnostic networks and laboratories to provide greater centralisation.<br>• Offering carrier screening and additional findings alongside diagnosis genome sequencing as a more logistically simple and streamlined approach to testing.<br>• Improved genetic risk stratification through family health history and other screening.<br>• Patient-facing and/or digital tools to streamline processes and clinical interactions (e.g., obtaining relevant family history information directly from a patient or family members by telephone, email, text or online, versus multiple forms or taking up time in a clinical appointment).<br>• Clear delineation of nursing roles and interventions in delivering genomic healthcare.<br>• Adaptive management philosophy: enables the collection and application of lessons and feeds this information into an engagement strategy. E.g., Structuring projects into portfolios enables the sharing of resources, processes, and learning, as well as sustainable implementation.<br>• Investigation to identify barriers or organisational obstacles.

**Data Management**<br><br>(18,20,21,33,34,37)	• Inconsistent modes for integrating genomic information into the EHR system.<br>• Insufficient data storage capacities.<br>• Lack of interoperability to make genomic results easily accessible and transferable within the EHR. Patients are unable to access their information and cannot share them with healthcare providers.	• Genomic testing and information accessible within EHR.<br>• Sharing data, tools, experience, and knowledge to create a global ''learning health system''.<br>• Digitisation of health data, prescriptions and healthcare billing to improve ease of access and engagement.
**Evaluation**<br><br>(16,24,26–28,34,35,47)		• Demonstration models enable the testing of implementation approaches.<br>• Investigation to identify barriers or organisational obstacles.<br>• Implementation of feedback loops.
**Collaboration**<br><br>(24,28,34,36–38,42,43,45,46)	• Lack of understanding of healthcare professional and researcher roles, responsibilities and accountabilities.<br>• The perception that innovative technology should be driven by clinicians rather than industry.	• Clinical leadership and clinician enthusiasm as facilitators of genomic implementation.<br>• Regular communication between investigators, clinicians and management among sites (via webinars, conference calls, and presentations) to solicit feedback and unify teams.<br>• Engagement of multidisciplinary teams across a range of conditions and expertise.<br>• Interdisciplinary clinics to manage patients with genomic conditions.<br>• Collaborative relationships between nurses, physicians, pharmacists, and clinical genetics professionals.<br>• International partnerships and knowledge sharing to promote a global 'learning health system' – essential for countries with an underdeveloped healthcare system.
